# Analysis and comparison of the trends in the burden of migraine in China and globally from 1990 to 2021, with a forecast to 2031

**DOI:** 10.3389/fneur.2025.1630720

**Published:** 2025-10-31

**Authors:** Wenjun Tang, Liyan Tang, Aiguo Li, Wei Zhang, Chaosheng Qin, Lixue Chen

**Affiliations:** ^1^Laboratory Research Center, The First Affiliated Hospital of Chongqing Medical University, Chongqing, China; ^2^Department of Clinical Laboratory, The First Affiliated Hospital of Guilin Medical University, Guilin, Guangxi, China; ^3^Emergency Department, Guilin Hospital of the Second Xiangya Hospital, Central South University, Guilin, China; ^4^Department of Anesthesiology, Guilin Municipal Hospital of Traditional Chinese Medicine, Guilin, China; ^5^Department of Anesthesiology, The First Affiliated Hospital of Guilin Medical University, Guilin, China

**Keywords:** migraine, GBD 2021, incidence, prevalence, disability-adjusted life years

## Abstract

**Background:**

Migraine is a prevalent neurological disorder in China and globally, imposing significant burdens on individuals and societies. The goal of this study was to characterize age- and sex-specific temporal patterns in migraine burden in China and worldwide from 1990 to 2021, focusing on incidence, prevalence, and disability-adjusted life years (DALYs).

**Methods:**

This retrospective population-based study utilized data from the Global Burden of Disease (GBD) Study in 2021 to analyze the characteristics of migraine burden in China and globally. The study focused on changes in incidence, prevalence, and DALYs. Joinpoint analysis was employed to calculate the average annual percentage change (AAPC) and the corresponding 95% confidence interval (95% CI) to assess trends in the burden of migraine. The Autoregressive Integrated Moving Average (ARIMA) model was utilized to predict the migraine burden for the next decade.

**Results:**

The worldwide age-standardized incidence rate (ASIR) of migraine rose from 1136.90/100,000 to 1153.20/100,000 between 1990 and 2021, whereas the ASIR in China grew from 917.35/100,000 to 975.61/100,000. Globally, the age-standardized prevalence rate (ASPR) rose from 14027.65/100,000 to 14246.55/100,000, while in China, it rose from 10948.52/100,000 to 11777.51/100,000. Female rates consistently exceed male rates across all age groups. Migraine incidence peaked in the 10–14 age group, while the prevalence and disability-adjusted life years (DALYs) peaked in the 30–34 age group for both females and males in 2021. The ARIMA model forecasts an upward trend in the ASIR and age-standardized DALYs rate (ASDR) for males in China over the next decade. Furthermore, the ASIR, ASDR, and ASPR for males globally, along with the ASIR and ASPR for females globally, are also anticipated to increase by 2031.

**Conclusion:**

Our findings indicate a rising burden of migraine in China and globally. Females are more susceptible to migraine in all age groups. The burden of migraine is age-related, with adolescents and youths facing a higher risk. More research is needed to determine the risk factors and illness patterns linked to migraine to facilitate early diagnosis, prompt therapies, and lessen patient burden, especially for females and adolescents.

## Introduction

Migraine is a debilitating neurovascular disorder characterized by recurrent unilateral pulsating headaches of moderate-to-severe intensity, which significantly impairs quality of life and constitutes a major global public health concern (1–3). The GBD 2021 estimates that 1.16 billion people worldwide are affected by migraine, with a prevalence of approximately 14% (95% UI, 12.9 to 15.2) (4, 5). This situation increasingly exposes a growing number of people to the threat of migraine, presenting challenges for society, which must address the rising burden associated with this condition as well as the pressures on healthcare systems (6). In fact, among all human diseases, migraine ranks second in terms of years lived with disability (YLDs) (7). Migraine is more prevalent among females, particularly in children and adolescents (8, 9). In 2019, the global incidence of migraine increased to 87.6 million (95% UI, 76.6 to 98.7), representing a 40.1% rise compared to 1990 (10).

As highlighted by the GBD, migraine burden has shown a significant upward trend from 1990 to 2021 (11). Migraine is a prevalent neurological disorder in China, significantly affecting quality of life (12). With a population exceeding 1.41 billion (20% of the global total), China has one of the highest numbers of migraine sufferers (13). Current GBD-based studies on migraine burden primarily focus on global or regional macro-level assessments (14–16), and recent work has estimated global trends, attributable risks, and future projections of migraine among adolescents (17). However, these studies rarely explore differences between individual countries and the global context, focusing instead on global perspectives or specific demographics and neglecting country-specific contexts. Despite growing medical attention to migraine prevalence in China—the world’s most populous nation—and existing related studies, further investigation is still needed (18, 19).

Our study aims to provide a comprehensive analysis of migraine trends—an understudied area for the Chinese population and globally. Using Joinpoint regression, we examine temporal patterns of migraine burden in China and worldwide from 1990 to 2021, exploring changes over 32 years through age and gender lenses. To forecast the next decade’s burden, we utilize GBD 2021 data with advanced statistical methods, decomposition analysis, and the ARIMA model. The goal is to assess global and Chinese migraine burdens to inform targeted prevention strategies and equitable resource allocation. Additionally, compared to pre-2021 studies, ours uniquely captures potential COVID-19 impacts, offering insights into how this global health emergency may affect migraine incidence, prevalence, and DALYs in China and globally.

## Methods

### Data source and disease definition

This analysis uses GBD 2021 migraine data, which provides epidemiological estimates of 371 diseases and injuries from 1990 to 2021. Migraine-related data were sourced from the Global Health Data Exchange (GHDx) platform and its tools^1^ (11). We extracted 1990–2021 GBD data on incidence, prevalence, and DALYs for China and the world—metrics essential for determining migraine impact. As the GBD 2021 data are publicly available, the institutional ethics committee granted an exemption, with no permission required. This study follows transparent and accurate health assessment reporting guidelines, with detailed data, methods, and statistical models available in previous reports (20). If a patient’s symptoms fit all five of the main diagnostic standards listed in the International Classification of Headache Disorders’ third edition (ICHD-3), they can be diagnosed with migraine. In the International Classification of Diseases (ICD), migraine is represented by codes 346–346.93 in ICD-9 and G43–G43.919 in ICD-10 (11).

### Statistical analysis

We extracted data from the GBD database about the incidence, prevalence, DALYs, and associated ASIR, ASPR, and ASDR for migraine in China and globally. We also acquired the crude DALY rate (CDR), crude incidence rate (CIR), and crude prevalence rate (CPR) for every age group. To evaluate the trend in disease burden, the AAPC was computed using Joinpoint software (21). A regression model was fitted to the logarithm of age-standardized indicators, which can be written as ln(y) = *α* + *β*x + *ε*, where x is the year and y is the corresponding age-standardized indicator. 100 × (exp(β) − 1) was used to get AAPC, and the model can also be used to determine the 95% CI. If the 95% confidence interval of the estimated AAPC is greater than 0, the age-standardized indicator indicates an increasing trend; if it is less than 0, a decreasing trend is observed; and if it includes 0, the trend is considered stable (22, 23).

Differentiating the time series data was the first step in the ARIMA modeling procedure. The best optimized model was selected based on the Akaike Information Criterion (AIC) using the auto.arima () function (24). Q-Q plots, autocorrelation function (ACF) plots, and partial autocorrelation function (PACF) plots were used to evaluate the distribution of the residuals for normality. The Ljung-Box test was then used to determine whether white noise was present in the residual sequence. Joinpoint 4.9 software (Statistical Research and Applications Branch, National Cancer Institute, United States) was used to perform the Joinpoint analysis. Stata 14.0 software was used to generate age-period-cohort models (StataCorp LP, TX, United States). The “forecast,” “tseries,” and “ggplot2” tools in R 4.1 software (R Core Team) were used to conduct ARIMA analysis and charting. Statistical significance was defined as a *p*-value of less than 0.05 (25).

## Results

### Description of the burden of migraine in China and worldwide

#### Incidence of migraine in China and globally

The cumulative rise in the incidence of migraine in China was 13.28%, from 11,518,098 cases (95% UI: 10,091,942–13,156,842) in 1990 to 13,047,221 cases (95% UI: 11,597,731–14,698,852) in 2021. Globally, the incidence increased by 42.03% from 1990 to 2021, from 63,496,591 cases (95% UI: 55,194,751–72,208,003) to 90,183,387 cases (95% UI: 78,857,600–101,838,163). In 1990, China’s ASIR was 917.35 (95% UI: 808.35–1036.95) per 100,000 people; by 2021, it had increased to 975.61 (95% UI: 862.32–1102.06) per 100,000 people. As of 2021, the ASIR was 1153.20 (95% UI: 1006.07–1304.49) per 100,000 people worldwide, up from 1136.90 (95% UI, 995.14–1287.77) per 100,000 in 1990. Additionally, between 1990 and 2021, the incidence rate of AAPC in China rose by 0.198% (95% UI, 0.189–0.207), whereas the global AAPC incidence rate grew by 0.046% (95% UI, 0.042–0.048) during the same period (Table 1).

**Table 1:** All-age cases and age-standardized incidence, prevalence, and DALYs rates and corresponding AAPC of migraine in China and globally in 1990 and 2021.

Location	Measure	1990 All-ages cases	Age-standardized rates per 100,000 people	2021 All-ages cases	Age-standardized rates per 100,000 people	1990–2021 AAPC
		*n* (95% UI)	*n* (95% UI)	*n* (95% UI)	*n* (95% UI)	*n* (95% CI)
China	Incidence	11,518,098 (10091942–13,156,842)	917.35 (808.35–1036.95)	13,047,221 (11597731–14,698,852)	975.61 (862.32–1102.06)	0.198 (0.189 −0.207)
	Prevalence	133,474,537 (114199444–153,482,598)	10948.52 (9428.76–12586.13)	184,752,280 (160836525–213,633,958)	11777.51 (10137.56–13538.56)	0.236 (0.224–0.247)
	DALYs	5,028,787 (767668–11,262,271)	412.97 (66.16–911.02)	6,988,196 (1133319–15,186,289)	443.65 (66.93–971.68)	0.233 (0.221–0.244)
Global	Incidence	63,496,591 (55194751–72,208,003)	1136.90 (995.14–1287.77)	90,183,387 (78857600–101,838,163)	1153.20 (1006.07–1304.49)	0.046 (0.042–0.048)
	Prevalence	732,564,463 (624559244–847,058,436)	14027.65 (12063.37–16078.07)	1,158,432,824 (995861966–1,331,312,506)	14246.55 (12194.12–16378.70)	0.051 (0.048–0.053)
	DALYs	27,412,196 (4076605–60,325,806)	526.76 (83.36–1145.92)	43,378,890 (6732642–95,079,454)	532.70 (80.57–1167.71)	0.036 (0.033–0.039)

DALYs, disability-adjusted life years; AAPC, average annual percentage change.

#### Prevalence of migraine in China and globally

In 1990, there were 133,474,537 (95% UI: 114,199,444–153,482,598) cases of migraine in China; by 2021, that number had risen to 184,752,280 (95% UI: 160,836,525–213,633,958), representing a cumulative increase of 38.42%. The prevalence increased by 58.13% globally, from 732,564,463 (95% UI: 624,559,244–847,058,436) in 1990 to 1,158,432,824 (95% UI: 995,861,966–1,331,312,506) in 2021. As of 2021, the ASPR in China was 11,777.51 (95% UI: 10,137.56–13,538.56) per 100,000 population, up from 10,948.52 (95% UI: 9,428.76–12,586.13) in 1990. As of 2021, the ASPR was 14,246.55 (95% UI: 12,194.12–16,378.70) per 100,000 people worldwide, up from 14,027.65 (95% UI: 12,063.37–16,078.07) per 100,000 in 1990. Furthermore, between 1990 and 2021, the prevalence of AAPC in China rose by 0.236% (95% UI: 0.224–0.247), whereas the global prevalence of AAPC increased by 0.051% (95% UI: 0.048–0.053) during the same period (Table 1).

#### DALYs of migraine in China and globally

In China, the DALYs attributed to migraine increased from 5,028,787 (95% UI: 4,767,668–5,126,271) in 1990 to 6,988,196 (95% UI: 5,133,319–7,186,289) in 2021, representing a 38.93% increase over the period. Globally, DALYs associated with migraine increased by 58.25% from 1990 to 2021. In China, the ASDR for migraine increased from 412.97 (95% UI: 66.16–911.02) per 100,000 population in 1990 to 443.65 (95% UI: 66.93–971.68) per 100,000 population in 2021. Similarly, the global ASDR increased from 526.76 (95% UI: 83.36–1,145.92) per 100,000 population in 1990 to 532.70 (95% UI: 80.57–1,167.71) per 100,000 population in 2021. Additionally, the AAPC in DALYs globally increased by 0.036% (95% UI: 0.033–0.039) from 1990 to 2021, whereas in China, the AAPC increased by 0.233% (95% UI: 0.221–0.244) over the same period (Table 1).

#### Joinpoint regression analysis of the burden of migraine in China and globally from 1990 to 2021

**Figure 1:**
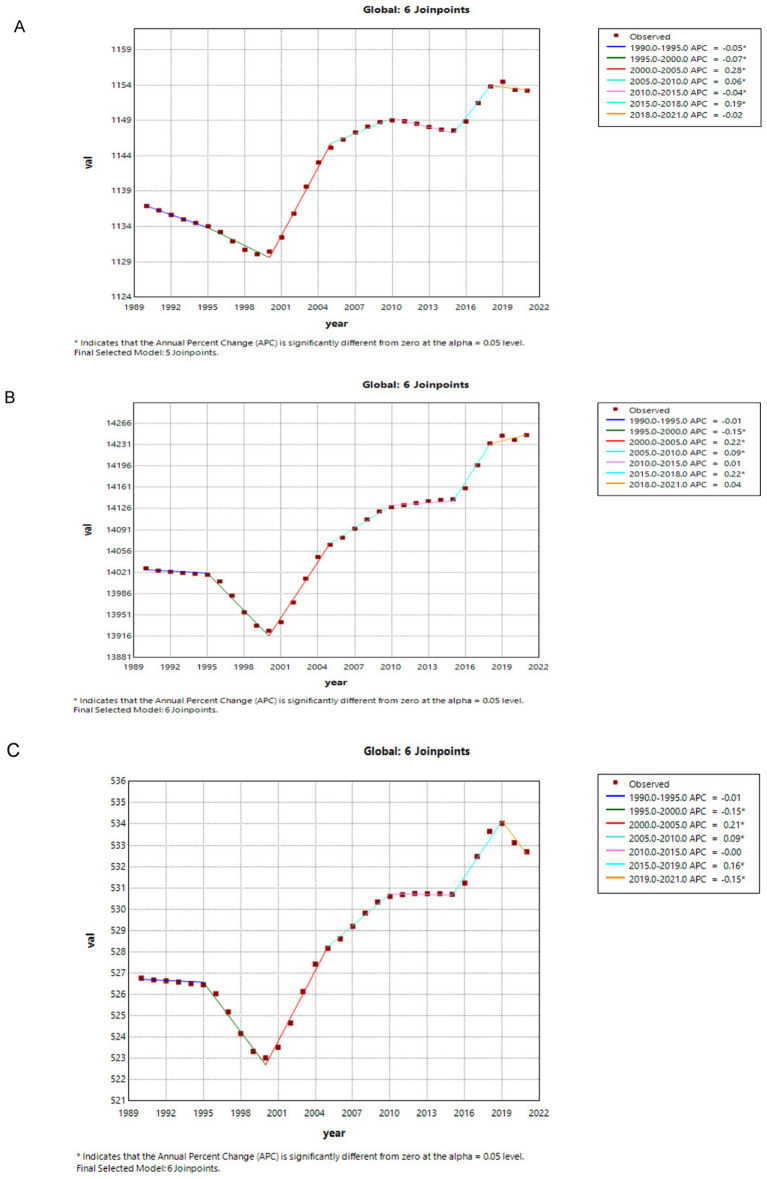
The APC of ASIR, ASPR, and ASDR of migraine in global from 1990 to 2021 (* means *p*-values < 0.05 and significant results). **(A)** ASIR; **(B)** ASPR; **(C)** ASDR. APC, annual percentage change; ASIR, age-standardized incidence rate; ASPR, age-standardized prevalence rate; ASDR, age-standardized DALYs rate; DALYs, disability-adjusted life years.

**Figure 2:**
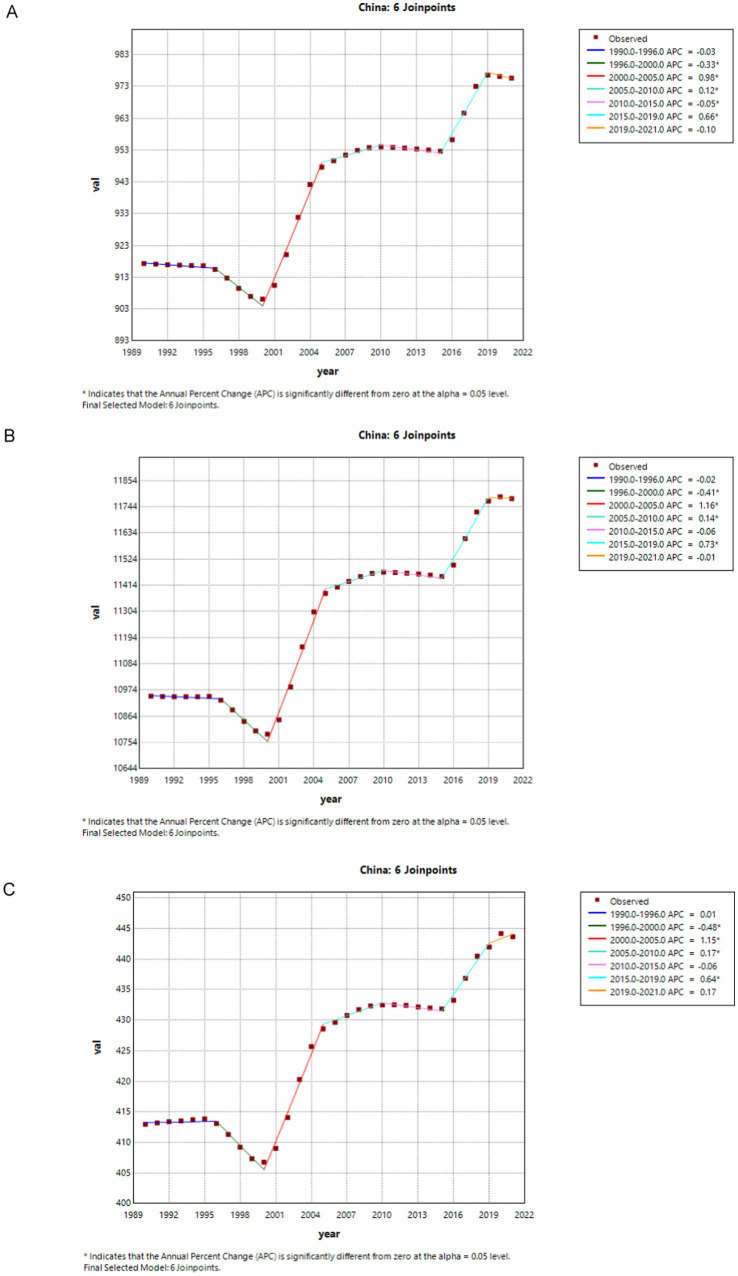
The APC of ASIR, ASPR, and ASDR of migraine in China from 1990 to 2021 (* means *p*-values < 0.05 and significant results). **(A)** ASIR; **(B)** ASPR; **(C)** ASDR. APC, annual percentage change; ASIR, age-standardized incidence rate; ASPR, age-standardized prevalence rate; ASDR, age-standardized DALYs rate; DALYs, disability-adjusted life years.

Figures 1, 2 show the results of a joinpoint regression analysis of DALYs, ASIR, and ASPR associated with migraine in China and globally between 1990 and 2021.

The migraine ASIR’s annual percentage change (APC) increased significantly worldwide between 2000 and 2005 and between 2015 and 2018 (2000–2005 APC = 0.28; 2015–2018 APC = 0.19, *p* < 0.05). Notably, a significant decline in ASIR was observed between 1990 and 2000, with subsequent fluctuations from 2010 to 2021, including a slight decrease during 2010–2015 and 2018–2021. For migraine ASPR, there was a significant decline between 1995 and 2000 (1995–2000 APC = −0.15, *p* < 0.05). However, an upward trend was evident from 2000 to 2021, with significant increases between 2000 and 2005 and again between 2015 and 2018 (2000–2005 APC = 0.22; 2015–2018 APC = 0.22, *p* < 0.05). Regarding migraine DALYs, a significant decline was observed between 1995 and 2000, and again between 2019 and 2021 (1995–2000 APC = −0.15; 2019–2021 APC = −0.15, *p* < 0.05). An upward trend was noted from 2000 to 2019, with significant increases during 2000–2005 and 2015–2019 (2000–2005 APC = 0.21; 2015–2019 APC = 0.16, *p* < 0.05) (Figure 1).

In China, both ASIR and ASPR for migraine showed significant declines from 1996 to 2000 (ASIR: 1996–2000 APC = −0.33, *p* < 0.05; ASPR: 1996–2000 APC = −0.41, *p* < 0.05), followed by a slight decrease from 1990 to 1996, and during 2010–2015 and 2019–2021. However, a significant upward trend was observed from 2000 to 2005 and from 2015 to 2019 (ASIR: 2000–2005 APC = 0.98; 2015–2019 APC = 0.66, *p* < 0.05; ASPR: 2000–2005 APC = 1.16; 2015–2019 APC = 0.73, *p* < 0.05). China’s migraine DALYs decreased significantly between 1996 and 2000 (DALYs: 1996–2000 APC = −0.48). From 2000 to 2021, there was an upward trend, with particularly noticeable increases during 2000–2005 and 2015–2019 (DALYs: 2000–2005 APC = 1.15; 2015–2019 APC = 0.64, *p* < 0.05) (Figure 2).

#### Trends in the burden of migraine in China and globally

**Figure 3:**
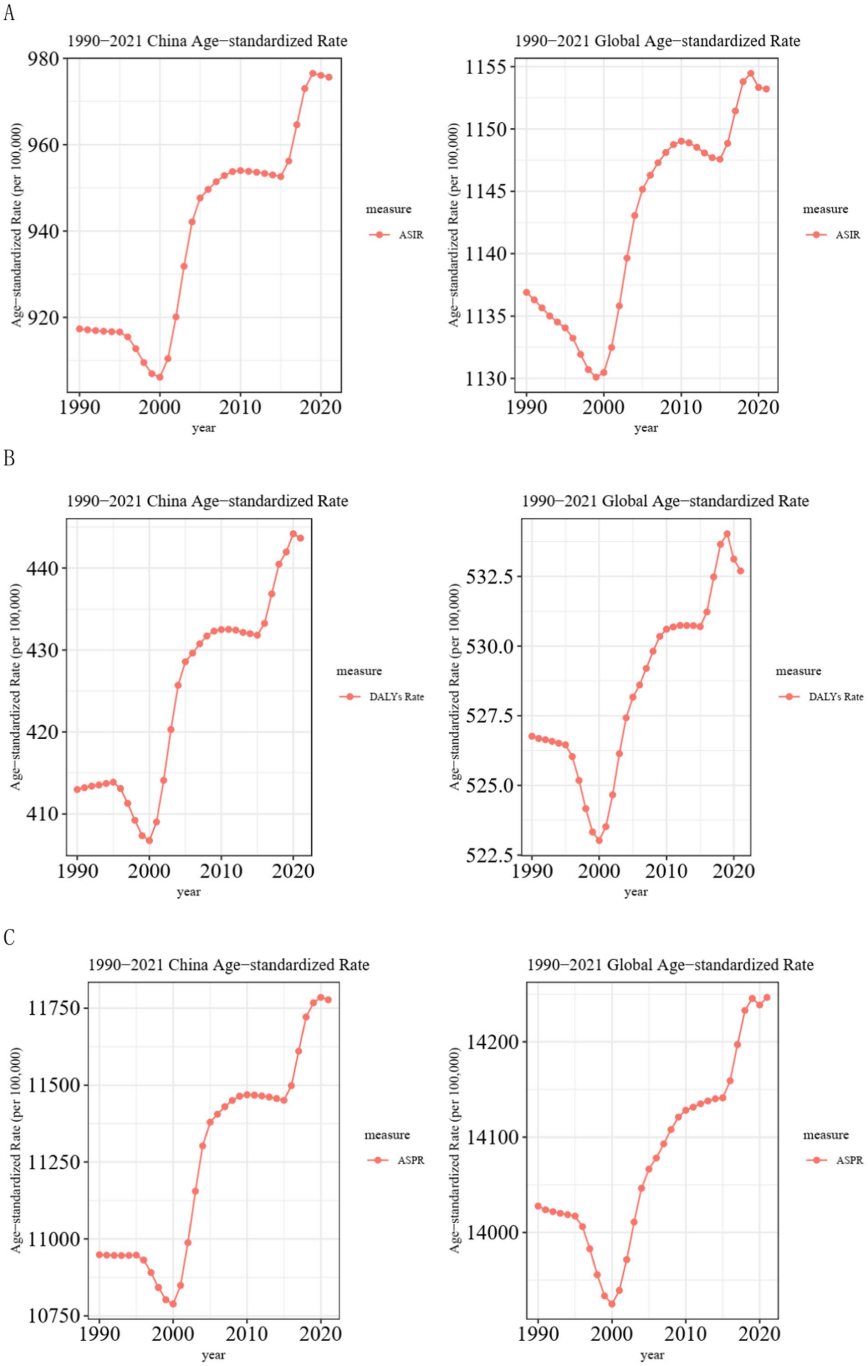
Trend comparison of ASIR, ASPR, and ASDR of migraine in China and globally from 1990 to 2021. **(A)** Trends of migraine ASIR in China and globally from 1990 to 2021; **(B)** Trends of migraine ASDR in China and globally from 1990 to 2021; **(C)** Trends of migraine ASPR in China and globally from 1990 to 2021. ASIR, age-standardized incidence rate; ASPR, age-standardized prevalence rate; ASDR, age-standardized DALYs rate; DALYs, disability-adjusted life years.

The ASIR for migraines showed a declining trend in China and globally between 1990 and 2000. The decline was more evident on a global scale. In China, there was only a slight decrease during the period from 1990 to 1995. However, it exhibited a remarkable upward trend from 2000 to 2005 and from 2015 to 2019, both in China and globally. Meanwhile, it demonstrated a slight downward trend from 2010 to 2015 and from 2019 to 2021 (Figure 3A).

The ASDR of migraines exhibited a downward trend worldwide from 1990 to 2000. In China, however, there was a slight increase from 1990 to 1995, followed by a notable decrease from 1995 to 2000. After 2000, both China and the global showed a significant upward trend. However, globally, there was a remarkable decline from 2019 to 2021 (Figure 3B).

In contrast, the ASPR for migraine in China stayed constant between 1990 and 1996 before experiencing a notable drop between 1996 and 2000. On the other hand, there was a slow fall from 1990 to 1996 and a sharp decline from 1996 to 2000 on a global scale. Following 2000, there was a notable increasing tendency in both China and globally. In China, there was a slight decline from 2010 to 2015 (Figure 3C).

#### Burden of migraine in different age groups in China and globally in 1990 and 2021

**Figure 4:**
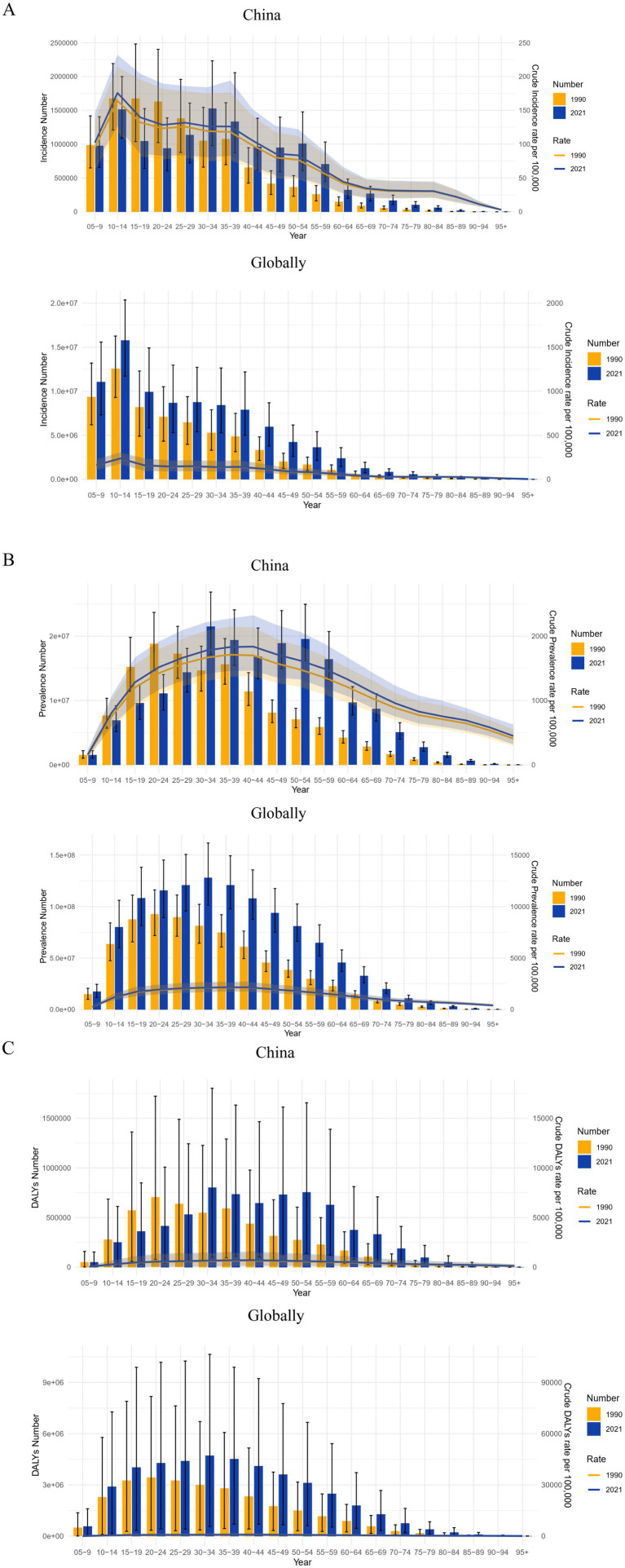
Comparison of the incidence, prevalence, and DALYs counts, along with their crude rates, by age group in China and globally from 1990 to 2021. **(A)** Incident cases and CIR; **(B)** Prevalent cases and CPR; **(C)** DALYs counts and CDR; Bar charts represent counts; lines represent crude rates. DALYs, disability-adjusted life years; CIR, crude incidence rate; CPR, crude prevalence rate; CDR, crude DALY rate.

A comparison of the incidence, prevalence, and DALYs of migraine in China and globally in 1990 and 2021 for various age groups, as well as the associated crude rates, is shown in Figure 4. According to incidence data, the age groups of 10–14 and 30–34 had the highest incidences of migraine in China in 2021. The 10–14 age group had the highest incidence worldwide in 1990 and 2021, closely followed by the 5–9 age group. The global incidence in 2021 showed an increase across all age groups compared to 1990, with similar trends noted for both prevalence and DALYs. In 1990, both in China and worldwide, the 20–24 age group had the highest prevalence and DALYs for migraine. DALYs and peak prevalence, however, moved to the 30–34 age range by 2021. Globally, migraine CIR rose from the 5–9 to 10–14 age group, then decreased in individuals aged 15 and older, with the 10–14 age group exhibiting the highest incidence.

#### Gender disparities in the burden of migraine in different age groups in China and globally

**Figure 5:**
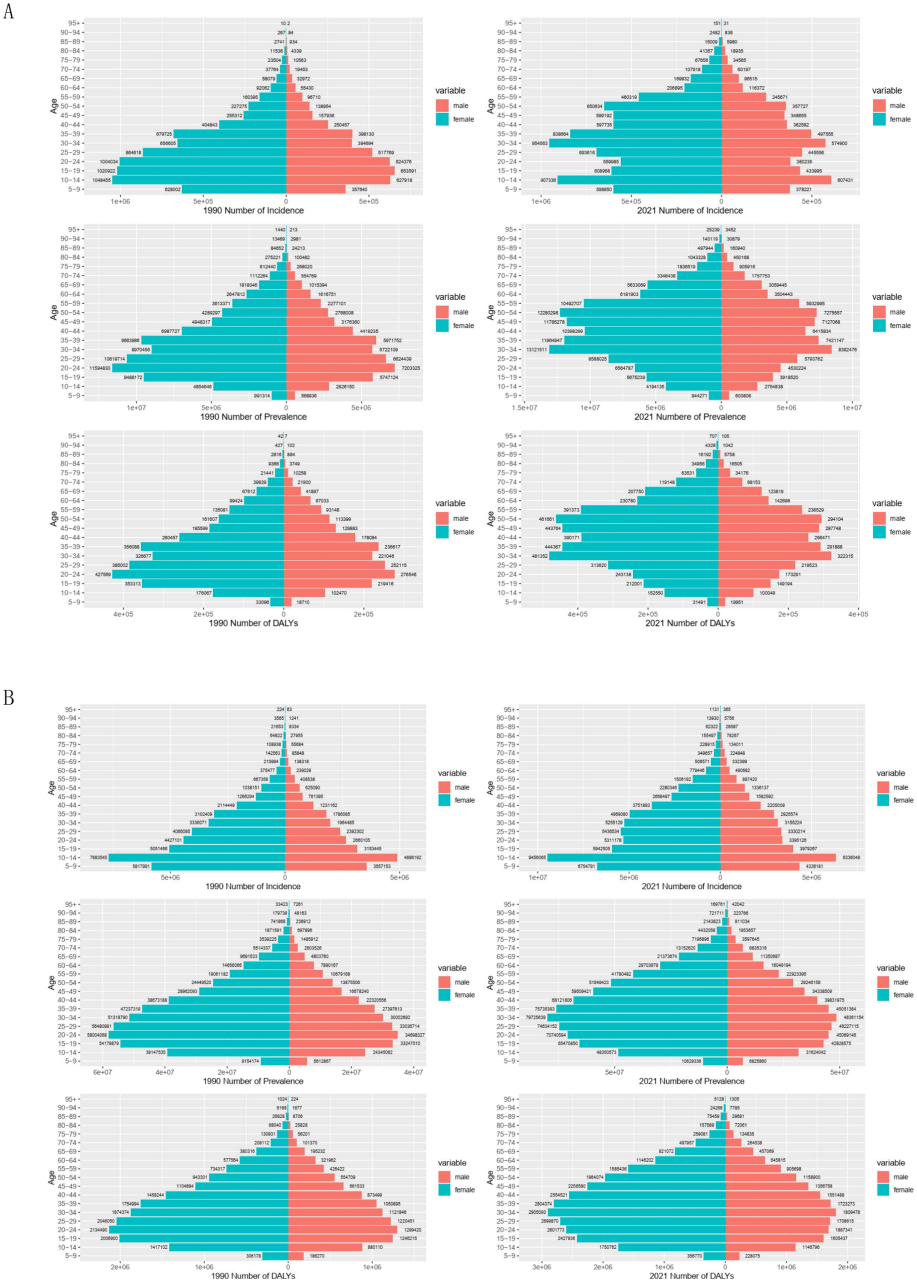
Comparison of the incidence, prevalence, and DALY of migraine in males and females of different age groups in China and globally in 1990 and 2021. **(A)** The incidence, prevalence, and DALY of migraine in China; **(B)** The incidence, prevalence, and DALY of migraine globally. DALY, disability-adjusted life years.

Figure 5 illustrates the incidence, prevalence, and DALYs associated with migraine across different age groups for both males and females in China and globally in 1990 and 2021. Notably, in all age groups, the values for females were significantly higher than those for males. Regarding incidence, in 1990, the highest incidence of migraine in China occurred in females in the 10–14 age group, while in males, it was observed in the 15–19 age group, followed by the 10–14 age group. By 2021, the peak incidence in females shifted to the 30–34 age group, with the 10–14 age group following closely. In males, the highest incidence was observed in the 10–14 age group, followed by the 30–34 age group. Globally, the highest incidence rates for both females and males were observed in the 10–14 age group, significantly surpassing those in other age groups.

According to 1990 prevalence data, from the 5–9 age group to the 20–24 age group, both males and females in China had an increase in migraine cases, with the highest prevalence in the 20–24 age group. A higher prevalence was observed among both sexes between the ages of 10 and 49, with a decline after age 25, reflecting a similar global trend. In China and globally, the peak prevalence for both males and females moved to the 30–34 age range by 2021. Worldwide, high prevalence was noted in individuals aged 10 to 54 years, while in China, the prevalence extended to the 10–69 age range. Across all age groups, DALYs trends were parallel to prevalence trends, with females experiencing higher DALYs than males. The 20–24 age group had the highest DALYs in 1990 for both males and females in China and globally. By 2021, both sexes’ peak DALYs had moved to the 30–34 age range.

The burden of migraine disease and age-standardized rates (ASIR, ASPR, and ASDR) for all age groups, including males and females, in China and worldwide between 1990 and 2021 are compared in Supplementary Figure S1. The data reveal a consistent upward trend in these rates over the years. Notably, the age-standardized rates for females were consistently higher than those for males across all years, with this increasing trend observed both globally and within China.

### Future burden of migraine

**Figure 6:**
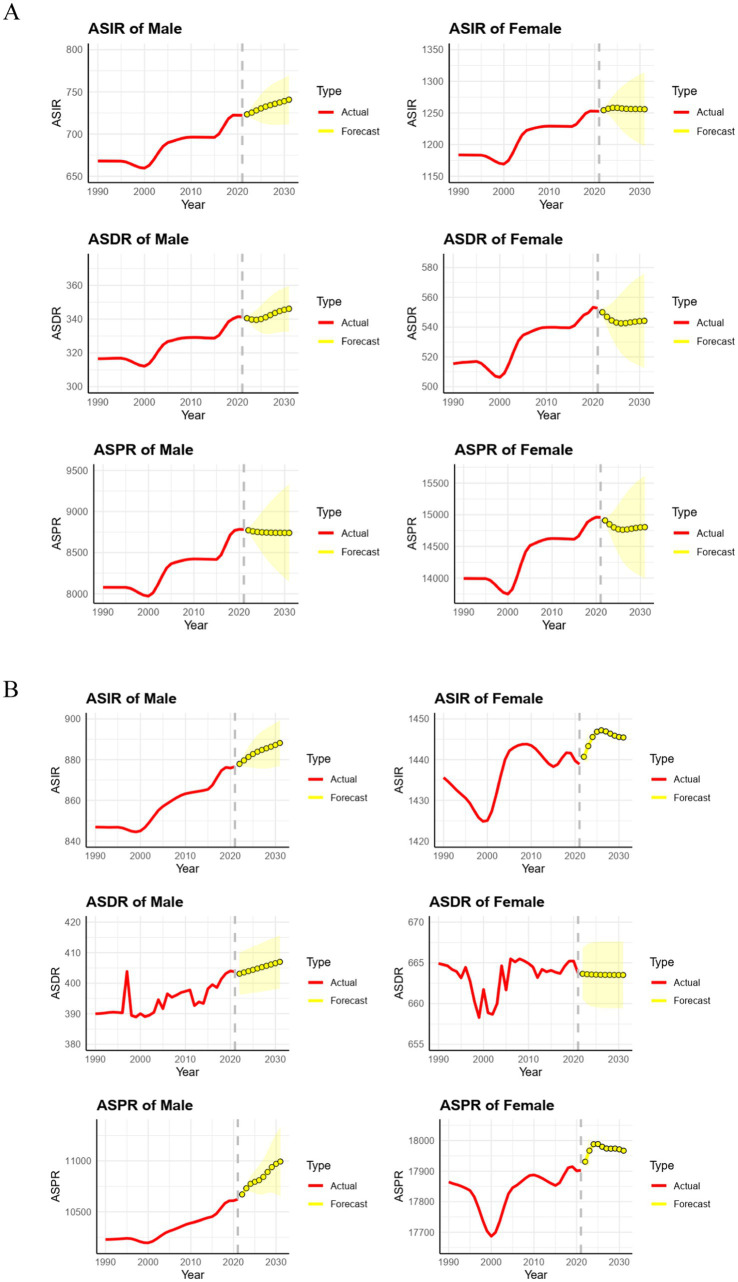
Predicted trends of migraine ASIR, ASDR, and ASPR in China and globally over the next 10 years (2022–31). Red lines represent the true trend of migraine ASIR, ASDR, and ASPR during 1990–2021; yellow dot lines and shaded regions represent the predicted trend and its 95% CI. ASIR, age-standardized incidence rate; ASDR, age-standardized DALY rate; ASPR, age-standardized prevalence rate; **(A)** Predicted trends of migraine ASIR, ASDR, and ASPR in China over the next 10 years; **(B)** Predicted trends of migraine ASIR, ASDR, and ASPR globally over the next 10 years. ASIR, age-standardized incidence rate; ASPR, age-standardized prevalence rate; ASDR, age-standardized DALY rate; DALY, disability-adjusted life years.

The forecasted trends in migraine ASIR, ASDR, and ASPR in China and worldwide are shown in Figure 6. Over the next ten years, both in China and globally, it is anticipated that the ASIR and ASDR for males will steadily rise. By 2031, the ASIR for males is expected to reach 741 per 100,000 (95% UI: 712–770) in China (Figure 6A) and 888 per 100,000 (95% UI: 877–899) globally (Figure 6B). Similarly, the ASDR for males is forecasted to reach 346 per 100,000 (95% UI: 333–360) in China and 407 per 100,000 (95% UI: 398–416) globally by 2031. For females, the ASIR of migraine is expected to increase globally, reaching 1,445 per 100,000 (95% UI: 1,424–1,467) by 2031. In contrast, the female ASIR in China is anticipated to remain relatively stable over the next decade. The ASDR for females is projected to decline slightly in both China and globally by 2031. The global ASPR for migraine is projected to increase over the next decade.

## Discussion

Our analysis of the trends in the burden of migraines in China and globally from 1990 to 2021 revealed a striking increase in ASIR, ASPR, and ASDR for migraine. This pattern emphasizes how urgently comprehensive public health initiatives are needed to combat rising cases of migraine (26). This study offers epidemiological insights regarding the burden of migraine in China and worldwide using data from the GBD 2021 and expanding on previous research. We used decomposition analysis and sophisticated statistical techniques to evaluate the effects of age, gender, and epidemiological variances, as well as the temporal patterns. Additionally, forecasts from the ARIMA method were included in our analysis, offering a forward-looking viewpoint to direct resource allocation and public health planning.

In this study, we used the GBD 2021 database to thoroughly assess migraine incidence, prevalence, and DALYs in China and globally over 32 years, comparing burdens by age and gender. Migraine showed a higher incidence in adolescents and females, highlighting unique challenges for individuals aged 10–39 and underscoring the need for age-specific support and targeted treatments to reduce long-term effects and improve quality of life. We built on prior findings on young adults and females while addressing other age groups (8, 17). Our study found a global incidence that peaked in the 10–14 age range for males and females in 2021. The incidence of 90,183,387 cases of migraine worldwide in 2021 is higher than the 87,648,969 cases reported by Fan et al. for 2019, indicating a continuing upward trend in migraine incidence (10). Controlling migraine incidence is key to reducing neurological disease burden, as its high prevalence—a chronic, often persistent condition—significantly contributes to global disability (27).

We observed an increase in the ASPR, with the global ASPR in 2021 reaching 14,246.55 cases per 100,000 people, in contrast to 14,027.65 cases per 100,000 people in 1990. Furthermore, we found that in 2021, the prevalence of migraine reached its peak in both males and females in the 30–34 age group, both globally and in China, with a trend toward younger ages of onset. With a population of 1.41 billion, China is the most populous country in the world and ranks second globally in the total number of migraine cases (10, 28). Compulsory education and frequent examinations for children, alongside academic admission and peer competition pressures in adulthood. During this period, exposure to adverse environmental factors and lifestyle behaviors increases the risk of migraine attacks.

Females exhibit greater susceptibility to migraine, with a marked gender disparity in prevalence. This aligns with global research showing higher migraine rates in women across all age groups and regions (29). Hormonal fluctuations—particularly estrogen’s influence on glutamate and serotonin systems involved in migraine pathophysiology—and elevated stress levels contribute to this difference. Additionally, gender disparities in healthcare access and help-seeking behavior may play a role: women are more likely to seek medical attention for migraines, potentially increasing diagnosis and reporting rates (30, 31). In China, cultural expectations may further hinder women’s migraine management, delaying treatment and reducing efficacy. Addressing these gender-specific challenges via tailored interventions is critical to improving outcomes and enhancing healthcare delivery and patient satisfaction.

The use of decomposition techniques and ARIMA analysis offers new viewpoints on the determinants and trends of migraine burden over time. These techniques make it possible to clarify the effects of population expansion, aging, and epidemiological changes more clearly than was possible with earlier research that concentrated on certain age groups. Looking toward 2031, projections indicate that without substantial intervention, the burden of migraine in China and globally will continue to rise (11). The ARIMA model forecasts a rising trend in the ASIR and ASDR for males in China over the next decade. Furthermore, the ASIR, ASDR, and ASPR for males globally, along with the ASIR and ASPR for females globally, are also anticipated to increase by 2031. This forecast highlights the necessity for proactive measures to reduce the effects of migraine.

It is imperative to monitor the trends in migraine prevalence closely. Future studies should focus on assessing the effectiveness of interventions and understanding the long-term implications of rising migraine rates on healthcare systems (32). The difference in access to healthcare and the availability of effective therapies is another important concern. Many individuals in China face barriers to receiving timely care, including limited awareness among healthcare providers and a shortage of specialized migraine treatments. Efforts to improve education and resources for both patients and healthcare professionals are essential to guarantee that people with migraines receive the proper treatment (33).

Public health initiatives aimed at raising awareness of migraine and its impact can encourage earlier help-seeking behaviors among patients and reduce the burden associated with chronic migraines. Collaborative efforts among government agencies, healthcare providers, and community organizations are essential for strengthening migraine management strategies. A multifaceted approach is required to combat the increasing burden of migraine in China and globally. Public health campaigns focused on increasing awareness of migraine, its triggers, and management strategies can help individuals better understand and manage the condition (34). To guarantee a thorough and well-coordinated response, these programs should address not only the general public but also educators, legislators, and healthcare professionals. Schools and universities can play a pivotal role in enhancing migraine awareness and supporting affected students (35).

Implementing stress management programs, promoting regular physical activity, and advocating for healthy sleep habits can help reduce the risk of migraine onset and improve overall health (36). Healthcare systems should prioritize training healthcare professionals to accurately diagnose and effectively treat migraines, particularly in regions with inadequate diagnosis (37). The gap in migraine care can be filled, particularly in underprivileged areas, by increasing access to telemedicine services and specialized migraine clinics. Additionally, research should concentrate on finding novel therapeutic targets and creating migraine medicines that are more efficient and widely available. Biomarker utilization and personalized medicine developments could help improve patient outcomes and optimize treatment plans.

### Limitations

This study has several notable limitations. First, our analysis is restricted to migraine, excluding tension-type headache—a prevalent primary headache disorder that frequently co-occurs with migraine and shares overlapping epidemiological patterns (38). Future research should integrate both to provide a more comprehensive understanding of headache-related public health burdens. Second, as a retrospective population-based study, it relies on GBD 2021 data, which may be prone to underreporting in regions with limited healthcare access. Additionally, joinpoint and ARIMA analyses are susceptible to temporal fluctuations in data quality. Future studies should explore risk factors driving incidence increases, validate projections with prospective data, and assess intervention effectiveness, particularly for adolescents and females.

## Conclusion

In China and globally, there was an overall increased trend in migraine incidence, prevalence, and DALYs between 1990 and 2021. There was a notable gender difference, with females experiencing higher rates than males. The occurrence of migraines is also significantly influenced by age, with teenagers and young people being more susceptible. Both in China and globally, migraine incidence peaked in the 10–14 age group, and the prevalence and DALYs peaked in the 30–34 age group for females and males in 2021. To provide early diagnosis, prompt, efficient therapies, and lessen the burden on migraine patients, particularly females and adolescents, more research is necessary to understand the risk factors and illness patterns of migraine.

## Data Availability

Publicly available datasets were analyzed in this study. This data can be found at: http://ghdx.healthdata.org/gbd-results-tool.

## Footnotes

^1^ http://ghdx.healthdata.org/gbd-results-tool

## Supplementary material

SUPPLEMENTARY FIGURE S1 Comparison of full-age cases and age-standardized rates of incidence, prevalence, and DALYs among females and males in China and globally from 1990 to 2021. **(A)** Incident cases and ASIR, prevalent cases and ASPR, and DALY counts and ASDR globally. **(B)** Incident cases and ASIR, prevalent cases and ASPR, and DALY counts and ASDR in China. Bar charts represent case counts, while lines represent age-standardized rates. ASIR, age-standardized incidence rate; ASPR, age-standardized prevalence rate; ASDR, age-standardized DALY rate; DALY, disability-adjusted life years.
