# Supplementary material for: Analysis and comparison of the trends in the burden of migraine in China and globally from 1990 to 2021, with a forecast to 2031
Source: Front Neurol. 2025 Oct 31;16:1630720. doi: 10.3389/fneur.2025.1630720 (PMC12621456; doi:10.3389/fneur.2025.1630720)

Fig. S1 Comparison of full-age cases and age-standardized rates of incidence, prevalence, and DALYs among females and males in China and globally from 1990 to 2021. (A) Incident cases and ASIR, prevalent cases and ASPR, and DALYs counts and ASDR globally. (B) Incident cases and ASIR, prevalent cases and ASPR, and DALYs counts and ASDR in China. Bar charts represent case counts, while lines represent age-standardized rates.

A


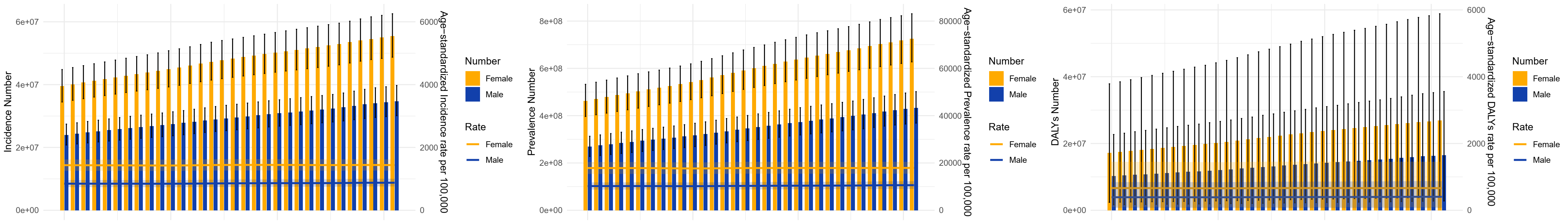


B


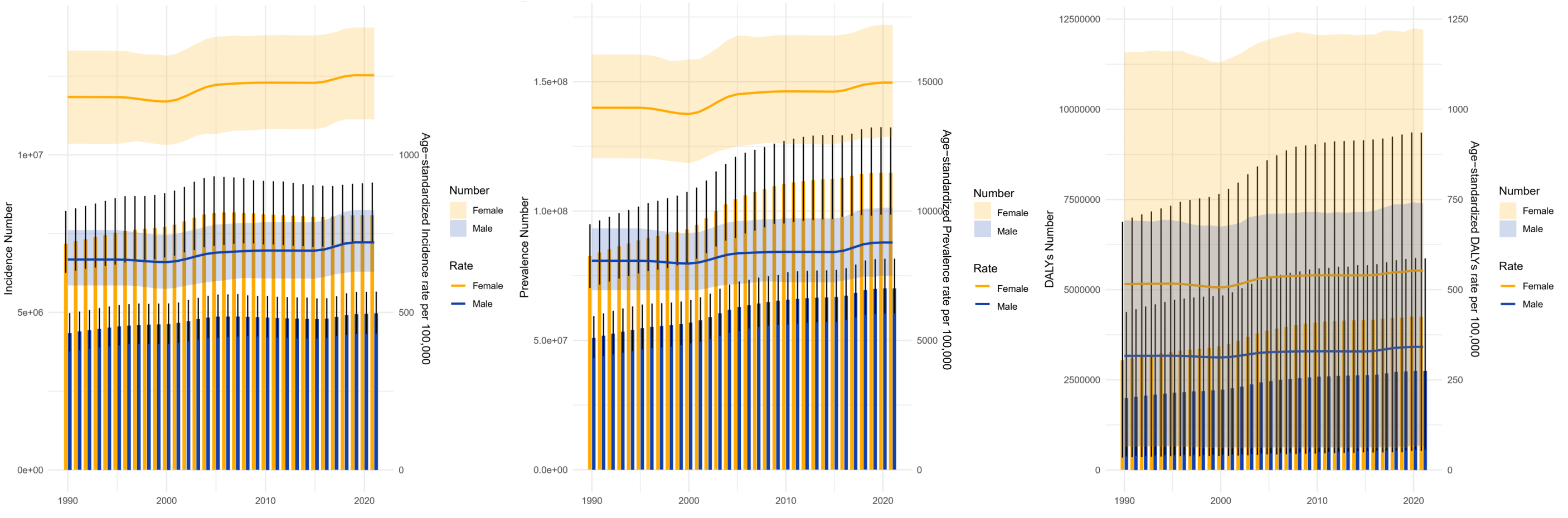

Supplement: SUPPLEMENTARY FIGURE S1 — Comparison of full-age cases and age-standardized rates of incidence, prevalence, and DALYs among females and males in China and globally from 1990 to 2021. (A) Incident cases and ASIR, prevalent cases and ASPR, and DALY counts and ASDR globally. (B) Incident cases and ASIR, prevalent cases and ASPR, and DALY counts and ASDR in China. Bar charts represent case counts, while lines represent age-standardized rates. ASIR, age-standardized incidence rate; ASPR, age-standardized prevalence rate; ASDR, age-standardized DALY rate; DALY, disability-adjusted life years. [file Supplementary_file_1.doc]
